# *Mannosidase 2*, *alpha 1* Deficiency Is Associated with Ricin Resistance in Embryonic Stem (ES) Cells

**DOI:** 10.1371/journal.pone.0022993

**Published:** 2011-08-23

**Authors:** Wei Wang, Christine Hale, Dave Goulding, Stuart M. Haslam, Bérangère Tissot, Christopher Lindsay, Stephen Michell, Rick Titball, Jun Yu, Ana Luisa Toribio, Raffaella Rossi, Anne Dell, Allan Bradley, Gordon Dougan

**Affiliations:** 1 The Wellcome Trust Sanger Institute, The Wellcome Trust Genome Campus, Hinxton, Cambridgeshire, United Kingdom; 2 Division of Molecular Biosciences, Imperial College London, South Kensington Campus, South Kensington, London, United Kingdom; 3 Cellular Toxicity Team, Biomedical Sciences Department, Porton Down, Salisbury, Wiltshire, United Kingdom; 4 School of Biosciences, University of Exeter, Exeter, Devon, United Kingdom; Strathclyde Institute of Pharmacy and Biomedical Sciences, University of Strathclyde, Royal College, Glasgow, Scotland, United Kingdom; French National Centre for Scientific Research - Université de Toulouse, France

## Abstract

Host gene products required for mediating the action of toxins are potential targets for reversing or controlling their pathogenic impact following exposure. To identify such targets libraries of insertional gene-trap mutations generated with a *PiggyBac* transposon in *Blm*-deficient embryonic stem cells were exposed to the plant toxin, ricin. Resistant clones were isolated and genetically characterised and one was found to be a homozygous mutant of the *mannosidase 2*, *alpha 1* (*Man2α1*) locus with a matching defect in the homologous allele. The causality of the molecular lesion was confirmed by removal of the transposon following expression of PB-transposase. Comparative glycomic and lectin binding analysis of the *Man2α1* (−/−) ricin resistant cells revealed an increase in the levels of hybrid glycan structures and a reduction in terminal β-galactose moieties, potential target receptors for ricin. Furthermore, naïve ES cells treated with inhibitors of the N-linked glycosylation pathway at the *mannosidase 2*, *alpha 1* step exhibited either full or partial resistance to ricin. Therefore, we conclusively identified *mannosidase 2*, *alpha 1* deficiency to be associated with ricin resistance.

## Introduction

Toxins are poisonous substances produced by a wide range of organisms in a variety of different forms including small molecules, peptides, proteins and larger order complexes which bind, enter and interfere with target cell functions by different mechanisms. While many toxins mediate physiological affects by targeting specific cellular components, the binding, entry and transport through a cell to their site of action is dependent on interactions with a variety of other cellular components. The identification and study of host factors which interact with toxins is important for a detailed understanding of how they disturb normal cellular physiology and to identify host cell components as potential targets for mitigating their effects.

Ricin is a heterodimeric lectin produced in the seeds of the castor oil plant, *Ricinus communis*. It is a type-2 toxin, having two chains, A and B. This toxin is extremely toxic and enters mammalian cells through the interaction of the ricin B chain with surface exposed β1–4 linked terminal galactose residues [1], [2]. Once inside the cell, ricin holotoxin is taken into early endosomes and a small fraction of the intoxicating protein then undergoes retrograde transport to the cytosol, via the endosomes, trans-golgi network (TGN) and endoplasmic reticulum (ER) [3]–[6], whilst the majority recycles to the cell surface. Once in the ER, the disulphide bridge between the A and B chain is reduced and the free A chain can be transported into the cytosol where it inactivates the 28S subunit of the ribosome. The process of crossing the ER is thought to involve the Sec61 translocon [7] in a fashion similar to that of misfolded proteins. Many steps in the process of uptake and retrograde transport of ricin are unknown and therefore of scientific interest.

Blm-deficient mouse ES cells have been developed as a platform for conducting phenotype-driven recessive screens. These cells have an elevated rate of loss of heterozygosity (LOH) approximately 20 fold higher compared with wild type ES cells. Following mutagenesis, the Blm-deficient background greatly facilitates the segregation of homozygous mutant clones, enabling genome wide recessive screens in the diploid mammalian genome. Using this genetic background we have successfully conducted screens for DNA mismatch repair genes and retroviral resistance [8], [9].

Here we describe a recessive screen designed to identify host cell components required for ricin toxicity. In contrast to our previous screens, *PiggyBac* transposon gene-trap vectors were used as insertional mutagens in Blm-deficient ES cells in place of retroviral vectors. The *piggyBac* transposon gene-trap vectors provide more comprehensive genome coverage and they have the additional advantage of seamless reversion by PBase compared with retroviral vectors [10].

In the screen described here, ricin resistant clones were directly selected from libraries of insertionally mutated Blm-deficient ES cells by exposing them to the toxin. A ricin-resistant clone with a homozygous mutation in the *mannosidase 2*, *alpha 1* (*Man2α1*) gene was identified confirming the utility of PB system for recessive genetic screens. Comparative glycomic analysis and glycosylation inhibition studies revealed that *Man2α1* deficiency altered the sugar spectrum on the ES cell surface. Immunogold imaging showed a reduction in ricin entry in *Man2α1* deficient cells. Thus the terminal β-galactose moieties are potential target receptors for ricin.

## Results

### Screening ES cell transposon libraries for ricin resistant mutants

**Figure 1:**
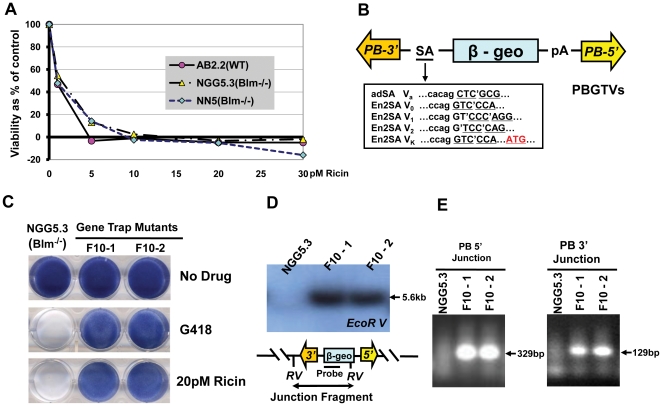
Mutagenesis and selection of ricin resistant mutants. A, establishing the selective dose of ricin. Viability as percentage of control calculated from neutral red staining of AB2.2, NN5 and NGG5.3 cells after exposure to different ricin doses. B, PBGTVs vector structure showing the position of the *PiggyBac* (PB) 3′ and 5′ inverted terminal repeats, the adenovirus (adSA) and the mouse gene *En2* (En2SA) splice acceptor, the polyadenylation site (pA) and the β-geo cassette. C, methylene blue staining of NGG5.3 cells or ricin-resistant mutant F10 clones -1 and -2 before and after treatment with G418 or 20 pm ricin. D, Southern blot of NGG5.3 or F10 clones -1 and -2 genomic DNA probed with the *β-geo* probe, a fragment of 5.6 Kb is visible in only F10 mutant clones -1 and -2. E, splinkerette PCR of the PB 5′ junction generates a fragment of 329 bp and the PB 3′ junction generates a product of 129 bp.

Libraries of ES cells with gene trap mutations were screened in an attempt to identify mutant cells with enhanced resistance to ricin. Prior to screening, a selective (lethal) dose of ricin for ES cells was established for the wild type parental cell line (AB2.2), the Blm-deficient feeder-dependent line (NGG5. 3) and a Blm-deficient mutant line adapted for growth in feeder-free conditions (NN5). A clonal survival assay was performed by exposing the ES cells to a range of ricin concentrations (1–30 pM) for 3 days and counting colonies after 10 days. The lethal dose of ricin for all cell lines was determined to be 10 pM after a 72 hour exposure (Fig. 1A).

A series of *PiggyBac* (PB) transposon-based gene-trap vectors (PBGTVs; Fig. 1B) containing an adenovirus splice acceptor (adSA) or a mouse gene *En2* splice acceptor (En2SA) and a β-geo gene-trap cassette flanked by the 5′ and 3′ PB terminal DNA repeats were used as the gene-trap vectors in this study. The *PiggyBac* transposon offers the advantage of being reversible, precise excision can be achieved by re-expressing the PB transposase [11], [12]. Previous studies [13] have reported that the 5′ PB terminal repeat has promoter activity. To avoid any possible influence of such an activity, the gene-trap cassette was placed in the opposite orientation relative to the 5′ PB terminal repeat. To maximize the coverage of the genome, five vectors (PBGTVa, V_0_, _1_, _2_ and V_K_) were used in which the coding sequence of β-geo is compatible with splicing from exons with different reading frames [10], [12], [14].

Independent co-transfections of the PB vectors with PBase were used to generate a gene-trap library in several sectors. Each sector of the library contained approximately 2,000 independent gene trap mutations, selected in G418. The clones constituting each sector were pooled and expanded through at least 14 cell doublings to facilitate the segregation of homozygous mutant clones and then they were selected with ricin. Each library sector generated varying numbers of resistant clones most of which were sister clones. From five sectors, twenty one independent insertion sites were analysed further. Initially the clones were screened to determine if they harboured homozygous mutations by cloning of the host-transposon junction fragments by splinkerette PCR. Two clones, named F10-1 and F10-2 which originated from the same pool, were examined further. In contrast to wild type Blm-deficient ES cell, the gene-trap mutant F10 clones -1 and -2 were still viable after exposure to 20 pM ricin, Figure 1C. The clonal relationship between clones F10-1 and -2 was investigated by Southern blot analysis using a β-geo probe and *Eco*RV to restrict the genomic DNA to reveal clone specific transposon-host junction fragments, Figure 1D. Both F10-1 and -2 showed a 5.6 kb fragment, suggesting that they are sister clones. This was confirmed by PCR analysis of the host-transposon junction fragments for both 5′ and 3′ PB ends, Figure 1E. Hence a single clone (referred to as F10) was taken forwards for further analysis.

### The ricin resistance gene-trap insertion maps to the *Mannosidase 2*, *alpha 1 (Man2α1)* gene

**Figure 2:**
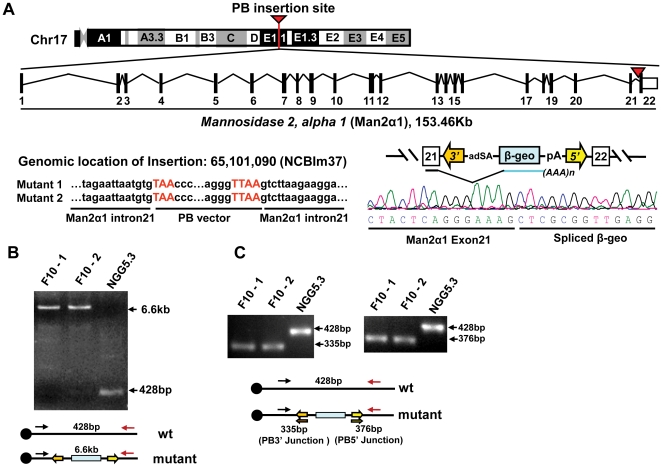
Insertion site of PiggyBac (PB) transposon into the mannosidase 2, alpha 1 gene. A, illustration of the insertion site in intron 21 of the *Man2α1* gene on murine chromosome 17. The precise insertion site and the exon 21-β-geo spliced product is shown. B, long range PCR on DNA from F10 mutant clones -1 and -2 or NGG5.3 generates a fragment of 6.6 kb for the mutants or 428 bp for the parent confirming homozygous insertion of the PB transposon. C, triple primer PCR confirms homozygosity by the generation of junction fragments corresponding to the PB 3′ end of 335 bp and the PB 5′ end of 376 bp in F10 mutant clones -1 and -2 compared to the parental NGG5.3 428 bp.

Sequence analysis of the host-transposon junction from clone F10 revealed that the PB transposon had inserted into intron 21 of the *Mannosidase 2*, *alpha 1* gene on chromosome 17, Figure 2A. This insertion was confirmed to be homozygous by long range PCR (LR-PCR) and triple primer PCR, Figures 2B and C. LR-PCR from the F10 clone generated a 6.6 kb product which included the whole PB transposon and 428 bp of flanking DNA while the PCR product from the NGG5.3 cell line was a 428 bp fragment amplified from the genomic locus, Figure 2B. The triple primer PCR on F10 genomic DNA generated a 335 bp fragment from the 3′ PB-host junction or a 376 bp fragment from the 5′ PB-host junction but a wild type fragment was not amplified. The same primer set only amplified the endogenous wild type 428 bp fragment in NGG5.3 (Fig. 2C).

### Reversion analysis

**Figure 3:**
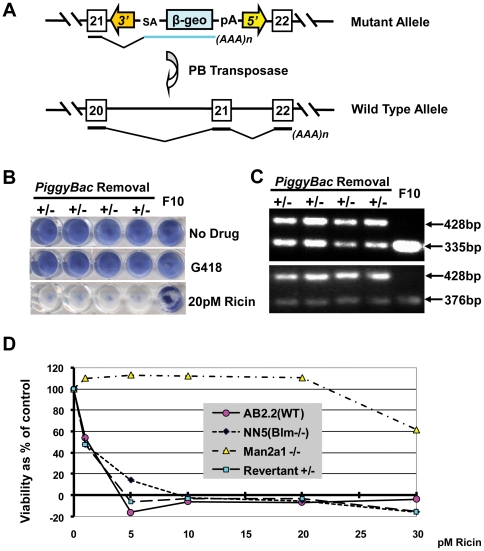
PBase reversion of mannosidase 2, alpha 1 mutation. A, illustration of the allele bearing the *Man2α1PiggyBac* insertion before and after reversion with PBase. B, methylene blue staining of four revertant heterozygous clones, and the original ricin resistant F10 clone, either before or after G418 or 20 pM ricin exposure. C, triple primer PCR demonstrating heterozygosity after reversion. D, viability as percentage of control calculated from neutral red staining of AB2.2, NN5, F10 *Man2α1* homozygous mutant and revertant cells after exposure to different concentrations of ricin (0 or 1–30 pM).

To establish causality between the insertion of the transposon and the ricin resistant phenotype, the transposon was designed to be revertible with PBase. The entire gene trap transposon cassette can be removed with PBase which precisely excises the transposon without leaving a footprint, Figure 3A.

The F10 cell line was transiently transfected with PBase and plated at clonal density to isolate revertant clones. These clones were isolated and sib-selected to test their sensitivity to ricin. Four out of 96 tested clones regained ricin sensitivity (Fig. 3B). Analysis of the PB-insertion site in the *Man2α1* locus revealed that all four resistant clones had lost the PB transposon from one copy of the locus, restoring one allele to its original wild type sequence and function (Fig. 3C). The ricin sensitivity of the heterozygous mutant clones was evaluated by exposure to a range of ricin concentrations. This revealed that the heterozygous clones were indistinguishable from wild type clones, illustrating that there was no dosage affect in the heterozygous state (Fig. 3D).

### Ricin resistance conferred by a glycosylation inhibitor

**Figure 4:**
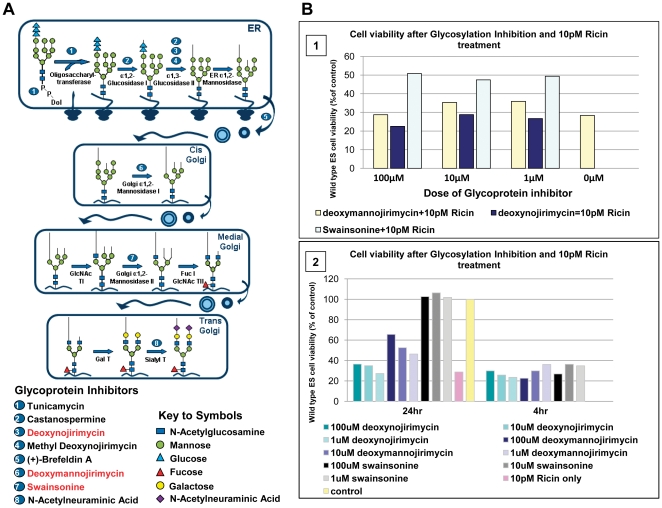
N-linked glycosylation pathway analysis. A, diagram indicating steps and key inhibitors (numbered 1–8) involved in processing core sugar structures. B1 assay demonstrating neutral red viability data after naïve ES cells were treated with glycosylation inhibitors deoxynojirmycin (#3 in fig. 4A), deoxymannojirimycin (#6 in fig. 4A) or Swainsonine (#7 in fig. 4A) at 1, 10 or 100 µM 4 hours prior to, and during, exposure to 10 pM ricin for 3 days. B2, a comparison of neutral red viability data after naïve ES cells were treated with glycosylation inhibitors either 24 hours or 4 hours prior to, and during, ricin exposure.

*Man2α1* is a key enzyme in the Golgi involved in N-linked glycosylation of proteins, a process which involves the sequential cleavage of glucose and mannose residues from the post-translationally added precursor structure, prior to the addition of alternative sugars, Figure 4A.

Well characterized inhibitors are available for steps in the glycosylation pathway, Figure 4A (numbered 1–8) [15]. In principle, inhibition of this pathway at an appropriate point could mimic aspects of the ricin resistance phenotype of the F10 mutant. We tested several inhibitors, deoxynojirimycin, deoxymannojirimycin and swainsonine (#3, #6 and #7 respectively, shown in red in Fig. 4A), which are believed to act prior to or on *Man2α1* in the pathway. Swainsonine is a reversible active-site inhibitor of Lysosomal α-mannosidase and blocks the processing of high mannose to complex type oligosaccharides. Its use 4 hours prior to and during ricin exposure protected approximately 10–25% of the exposed cells against ricin over and above inhibitor-free cells, whilst the remaining two inhibitors deoxynojirimycin and deoxymannojirimycin were less effective at all concentrations (Fig. 4B 1). To ensure that all cells were fully exposed to the inhibitors, pre-treatment was extended to 24 hours prior to ricin intoxication and this led to full protection by Swainsonine (Fig. 4B 2). However, deoxymannojirimycin still only exhibited partial protection (35% survival above that of ricin alone level) whilst deoxynojirimycin continued to afford little protection. The former is a specific glucosidase inhibitor of the trimming glucosidases I and II, enzymes that sequentially remove the three glucose residues from the precursor Glc_3_Man_9_-GlcNAc_2_ in N-linked glycan biosynthesis whilst the latter is a specific α-mannosidase I inhibitor that blocks conversion of high mannose to complex oligosaccharides. If the block in processing of N-linked glycans only was the direct causality of ricin resistance, inhibition of all steps prior to the action of mannosidase 2, alpha 1 should have been effective at inducing a level of ricin resistance. The fact that only Swainsonine protected fully indicates the importance of this step in the generation of the ricin receptor. Thus, it is possible that *Man2α1* is involved in other cellular processes such as the retrograde transport of mis-folded proteins, in a similar manner to *mannosidase 1 alpha*, potentially explaining why only Swainsonine consistently confers ricin resistance to naïve cells.

**Figure 5:**
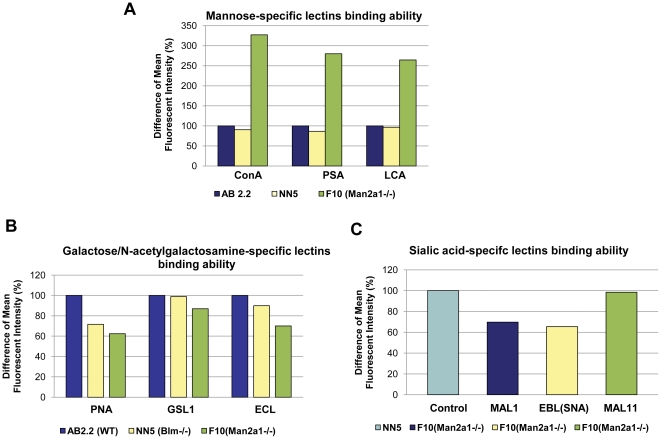
Mannose, galactose and sialic acid specific lectin binding analysis. Histograms of differences in Mean Fluorescence Intensity of lectin binding to either AB2.2, NN5 or ricin resistant F10 cells. A, Mannose specific lectins ConA, PSA and LCA represent *Concanavalin A*, *Pisum sativum agglutinin* and *Lens culinaris agglutinin* respectively. B, Galactose specific lectins PNA, GSL1 and ECL represent *Peanut agglutinin*, *Griffonia (Bandeiraea) simplicifolia lectin 1* and *Erythrina cristagalli lectin* respectively. C, Sialic acid specific lectins MAL1, EBL (SNA) and MAL11represent *Maackia amurensis lectin 1*, *Sambucus nigra lectin* and *Maackia amurensis lectin 11* respectively.

To confirm the impact of the mutation on surface sugars, flow cytometric analysis was conducted of cells incubated with a range of different FITC conjugated lectins. The F10 (*Man2α1*-deficient) line exhibited a 2.5–3 fold higher level of binding to lectins with specificity for mannose (Fig. 5A) than wild type controls. In contrast, lectins with specificity for either galactose or sialic acid, moieties added to the core sugar post mannosidase 2, alpha 1 action, were found to have reduced binding (Figs. 5B and 5C respectively), with respect to the control, in the ricin resistant F10 line.

### Immunogold imaging of ricin resistant cell line

**Figure 6:**
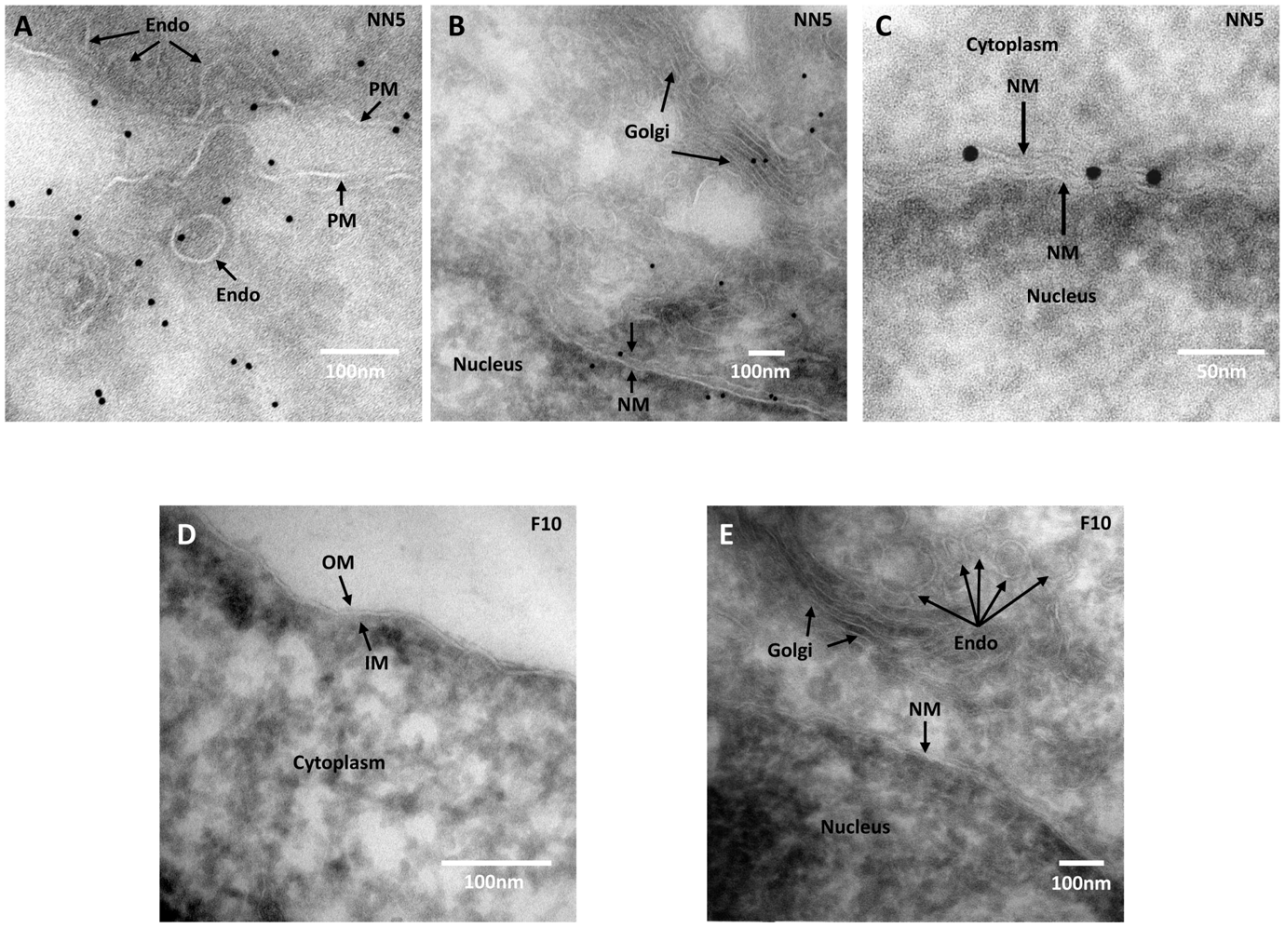
Anti-ricin immunogold staining of either NN5 or ricin resistant F10 cells. A–C, images showing ricin particles located in various sites within NN5 cells treated with 20 pM ricin for 1 h at 37°C. D and E ricin-resistant F10 cells exposed to 20 pM ricin and processed for anti-ricin staining at the same time. NM, PM, Endo, OM and IM represent nuclear membrane, plasma membrane, endosome, outer membrane and inner membrane respectively (small arrows). The nucleus, cytoplasm and Golgi are also indicated.

To investigate how the alterations in surface glycosylation patterns revealed by lectin binding might be affecting ricin resistance, anti-ricin immunogold staining was conducted on cells exposed to ricin. After treatment with 10 pM ricin for 1 hour, control NN5 cells exhibited positive staining of the toxin throughout the cell. Ricin was demonstrated to be distributed on the plasma and nuclear membranes (Figs. 6A, B and C). The F10 ricin resistant cells exhibited no detectable staining (Figs. 6D and E), indicating that reduced levels of surface exposed hybrid N-glycans may be responsible for a reduced level of ricin binding and a corresponding ricin resistance phenotype.

### Glycomic analysis

**Figure 7:**
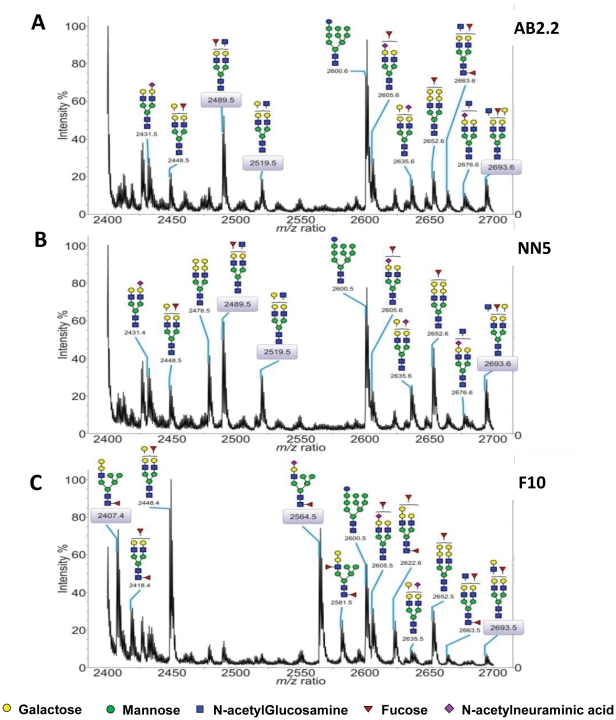
MALDI-TOF MS profiling of AB2.2, NN5 and F10 N-glycans (mz 2400–2700). The expanded region (m/z between 2400 and 2700) is shown on the panels A, (AB2.2), B, (NN5) and C, (F10). N-glycans were permethylated prior being subjected to MS analysis and m/z values correspond to [M+Na]^+^ ions. Scheme assignments are based on the precise fit between composition calculations and the *m/z* (*z* = 1) ratio of the molecular ions detected. These schemes represent the most likely structures taking into account the biosynthetic pathways and the enzyme repertoire of murine cells and selected MS/MS analyses. Sugar symbols are those employed by the Consortium for Functional Glycomics. Circles represent hexoses (yellow: galactose, green: mannose), blue squares represent N-acetylglucosamine, red triangle: fucose, purple diamonds represent N-acetylneuraminic acid.

**Figure 8:**
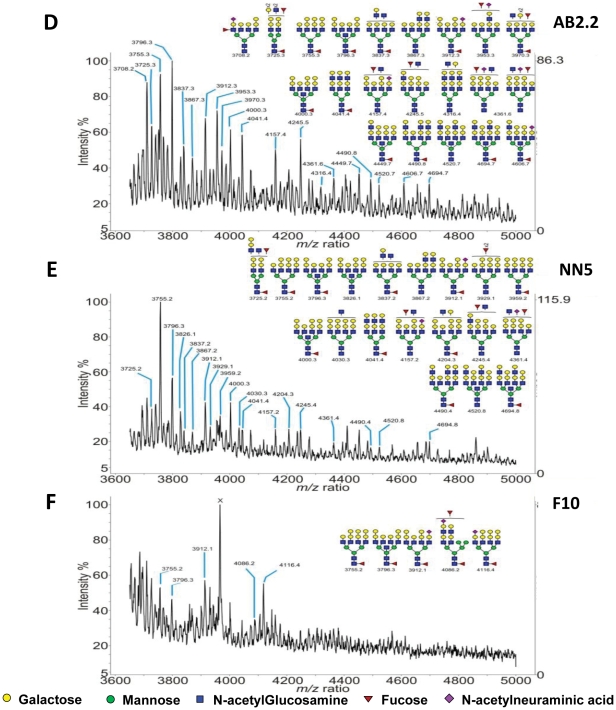
MALDI-TOF MS profiling of AB2.2, NN5 and F10 N-glycans (mz 3650–5000). The expanded region (m/z 3650–5000) is shown on the panels D, (AB2.2), E, (NN5) and F, (F10). N-glycans were permethylated prior being subjected to MS analysis and m/z values correspond to [M+Na]^+^ ions. Scheme assignments are based on the precise fit between composition calculations and the *m/z* (*z* = 1) ratio of the molecular ions detected. These schemes represent the most likely structures taking into account the biosynthetic pathways and the enzyme repertoire of murine cells and selected MS/MS analyses. Sugar symbols are those employed by the Consortium for Functional Glycomics. Circles represent hexoses (yellow: galactose, green: mannose), blue squares represent N-acetylglucosamine, red triangle: fucose, purple diamonds represent N-acetylneuraminic acid.

The parental and F10 cells were subjected to glycomic analysis (N- and O-glycans, glycosphingolipids) using MALDI-TOF MS, Figure 7. Apart from the high mannose structures, the glycan profiles of mutant and control cell types are similar (Supplementary Figures S1 and S2). However, two regions of these profiles exhibit some clear differences. It has been demonstrated that relative quantitation based on signal intensities of permethylated glycans analyzed by MALDI-TOF MS is a reliable method, especially when comparing signals over a small mass range within the same spectrum [16]. In the region m/z 2400 to 2700 (Fig. 7 A, B and C), the relative intensities of peaks such as m/z 2489, 2519 and 2693 which would have terminal β-linked galactose are all reduced in Mannosidase 2, alpha 1-deficient F10 cells (relative % intensities in AB2.2 cells 42/18/14, relative % intensities in NN5 cells 49/29/24 and relative % intensities in F10 cells 10/5/6). Additional F10 peaks at m/z 2407 and 2564 are also observed, which are not detectable in the profiles of the control cell lines. These correspond to hybrid type N-glycan structures on which only one mannosylated arm has been trimmed down and extended, the other arm remaining high-mannose like. In the second region of interest and at high m/z values (Fig. 8 D, E and F, Supplementary Figures S1 and S2), the F10 mutant exhibits less high molecular weight structures than the control. These data suggest that the complex binding specificity of ricin does not simply reflect the level of terminal galactose.

The O-glycan profiles (Supplementary table S2) exhibit very simple patterns with major structures being mono- and disialylated core-1. The samples do not show any major differences suggesting that the *Man2α1* mutation does not affect the O-glycosylation biosynthetic pathways. The polar and non polar species of glycolipids were also examined and also did not reveal any differences between the F10 *Man2α1*-deficient and control cell lines, revealing that this mutation did not affect the glycosphingolipid pathways either (Supplementary table S3).

## Discussion

The action of ricin on susceptible cells leads to rapid cell death induced by the inactivation of the 28S rRNA of the 60S ribosome subunit [17]. Many reviewers have alluded to the mechanisms of uptake of ricin but acknowledge a lack of full understanding. We have successfully conducted a genetic screen in ES cells to identify a gene, the mutation of which confers resistance to ricin intoxication. The ricin-resistant clone identified in this screen resulted from a homozygous mutation in the *Mannosidase 2*, *alpha 1* gene, which encodes a Golgi enzyme involved in N-linked glycosylation. The enzyme hydrolysis of the terminal α1–3 and α1–6 linked mannose residues of the GIcNAcMan5GlcNAc2 intermediate gives rise to the oligosaccharide GIcNAcMan3GlcNAc2. This represents one of the committal steps in the biosynthesis of complex N-glycans which will carry the terminal β-linked galatose residues which are recognised by ricin. The libraries used for this screen were generated in Blm-deficient ES cells which facilitates the segregation of homozygous mutants from pools of single allele mutations. This genetic background has supported several successful screens [8], [9], [18]. The mutation libraries used for this screen were generated using the *PiggyBac* transposon to insert a conventional gene-trap cassette into the ES cell genome [10]. One advantage of *PiggyBac* over other insertional mutagens is the fully revertible nature of the mutations, since re-expression of PBase seamlessly removes the *PiggyBac* transposon from the genome. The ricin-resistant mutant identified here could be reverted to a sensitive state by excision of the PB gene-trap transposon from one of the two copies of the *Man2α1* gene.

Our screen for ricin-resistant mutants from a *PiggyBac* gene-trap ES cell library has provided direct access to a gene required for ricin intoxication. To further our understanding of the mechanism of resistance we investigated the cell surface lectin binding properties and also conducted glycomic analyses on the mutant clones. As a confirmation of the direct involvement of *Man2α1* in ricin resistance, we treated naïve ES cells with inhibitors of N-linked glycosylation and found that Swainsonine conferred significant resistance to ricin exposure.

Meager and colleagues [19], have previously described ricin resistant (RicR) clones of baby hamster kidney cells. A selection of these proved to be resistant due to an inability to bind ricin. Metabolic labelling revealed that these clones express only hybrid and high mannose-type N-linked oligosaccharides rather than the complex sugars found in the parental BHK cells [20], [21]. Use of the mannose specific lectin, *Concanavalin A*, indicated that mannose residues were more abundant on those cells. Some of the RicR cells were deduced to be deficient in *Man2α1* activity but the causal defect was never genetically confirmed [22]. These data closely matched our observations on the ricin-resistant ES cell clone identified in our study which exhibited greatly increased binding of three different mannose specific lectins (*Concanavalin A*, *Pisum sativum agglutinin* and *Lens culinaris agglutinin*).

*Mannosidase 2*, *alpha 1* is known to cleave the N-linked GIcNAcMan5GlcNAc2 intermediate to complete the mannose trimming reactions and gives rise to the oligosaccharide, GIcNAcMan3GlcNAc2. The enzyme is mostly located in the medial-cisternae of cells but its distribution can vary with cell type [23]. The results from our glycomic analyses demonstrate that there are alterations in the hybrid and complex N-glycans released from our ricin-resistant clones. The high levels of residual complex glycans, still present in the F10 cells, indicates some redundancy in the complex N-glycan biosynthetic pathway. There is evidence of a complementary enzyme, alpha mannosidase llX [24] which also resides in the golgi and which can perform similar trimming but not to the extent of *Man2α1*. Mice deficient in both enzymes are mostly non-viable beyond the 18.5 d embryonic stage indicating the importance of N-linked glycosylation in embryo development [24], [25].

A recent publication has described an isolate, Lec36, of the human embryonic kidney HEK293T cell line, which has been identified as ricin resistant following ethyl methanesulfonate mutagenesis [26]. By nucleotide sequencing, the causal defect was linked to separate mutations on both alleles of *Man2α1*, one due to a point mutation at the active site and the second an in-frame deletion of twelve nucleotides. However, chemical mutagenesis can generate an array of unlinked and unmapped mutations that can contribute to a mutant phenotype. These data strongly support the results demonstrated in our homozygous gene-trap mutagenesis system and confirms the usefulness of screening mutation libraries in undifferentiated ES cells.

Immuno-gold imaging for ricin binding in the *Man2α1*-deficient ES cell clones failed to identify ricin bound to these cells. This suggests that the mechanism of resistance is related to the structure of the N-linked glycoproteins on the cell surface, which could be caused by loss of specific or general ricin-binding moieties on the cell surface.

In conclusion, we emphasise the usefulness of the *PiggyBac* gene-trap transposon system in Blm-deficient ES cells to generate mutants and directly identify genes required for action of toxins.

## Materials and Methods

### Cell culture

#### AB2.2, NN5 and NGG5.3 ES cell lines

These ES cell lines have been described previously [8]. The NN5 and NGG5.3 ES cell lines are Blm-deficient. The AB2.2 and NN5 cells were maintained on gelatin coated plastic in Glasgow's minimal essential medium (GMEM) supplemented with 10% foetal bovine serum, 2 mM L-glutamine, 1 mM sodium pyruvate, non-essential amino acids, 10^−4^ M β-mercaptoethanol and 500–1000 units of leukocyte inhibitory factor (LIF)/ml. NGG5.3 ES cells were maintained as described previously [27]. Briefly, ES cells were maintained on gamma-irradiated SNL76/7 feeder cell layers in Dulbecco's modified Eagle's medium supplemented with 15% foetal bovine serum, 2 mM L-glutamine, 50 units/ml penicillin, 40 µg/ml streptomycin, and 10^−4^ M β-mercaptoethanol. Cells were cultured at 37°C with 5% carbon dioxide.

#### Ricin resistant F10 cells

Clones arising from exposure to ricin were grown on SNL76/7 feeder cells until large enough to pick into 96 well plates also bearing feeder layers. The clones were expanded through 24 and 6 well plates until sufficient numbers were obtained to freeze down and prepare DNA for PCR analysis.

#### Adaptation of F10 to feeder-free conditions

To allow comparative experiments to be performed, feeder dependent F10 cells were trypsinised and washed once in NN5/AB2.2 medium prior to plating into gelatinized 90 mm dishes at a 1 in 8 dilution. Subsequent sub-culturing was performed every two days at the same dilutions until all feeder cells were depleted.

### Gene-trap vectors construction

The *PiggyBac* (PB) transposon vector containing 313 bp of the 5′ inverted terminal DNA repeat (TR) and 235 bp of 3′ inverted TR was provided by Dr. Xiaozhong Wang (North Western University). The PB5′ and PB3′ inverted repeats were PCR amplified and cloned upstream and downstream from the adSA- or En2SA-β-geo gene-trap cassette and named PBGTVs.

### Generation of gene-trap mutations

A single-cell clone of NGG5.3 ES cells was isolated and expanded to minimize background mutations. Five million cells were co-electroporated with 1 µg of gene-trap vector and 20 µg of a PBase expression plasmid pCAGGS-PBase, in which the native version of *PiggyBac* transposase coding sequence is under the control of the CAG promoter. After electroporation the cells were plated onto 90-mm feeder plates and G418 selection (180 mg/L active ingredient) was initiated 24 h later and continued for 8 days until individual ES cell clones were visible. The G418 resistant clones within each plate were pooled and expanded for 4 days before initiating ricin selection.

### Isolation of ricin resistant clones

Libraries of NGG5.3 cells with gene-trap mutations (10^6^ cells per 90 mm plate of feeder cells) were exposed to 20 pM ricin for 3 days; the plate was rinsed and maintained with normal medium over the next 7 days. Resistant colonies were grown until they were visible and picked into 96 well feeder plates.

### Neutral red and methylene blue assay for determining cell viability

NN5, AB2.2, NGG5.3 and F10 ricin resistant cells were plated at 10^5^ cells per well in 24 well plates and exposed to a range of ricin concentrations for 48 h. The cells were then mixed with 0.3% neutral red, incubated for a further 1 h and washed gently with phosphate buffered saline (PBS) for 1 h. After removal of the PBS, remaining viable cells were solubilised in a 1% glacial acetic acid/49% H_2_0/50% ethanol solubilisation buffer and values determined at OD 490 nm. As an alternative method, viable cells were directly stained in the plates with 2% methylene blue in 70% ethanol for 10 minutes and then washed extensively in tap water.

### Isolation of the transposon–chromosome junction

Isolation of the transposon–chromosome junction was performed using the splinkerette-PCR method as described [28]. The protocol was modified for use with ES cells on 96- or 24-well plates. Briefly, genomic DNA was isolated from ES cell colonies on 96- or 24-well plates; 2–4 µg DNA was digested with *Sau3A*I and ligated with the corresponding splinkerette adaptors HMSp–Sau3AI (generated by annealing splinkerette oligos HMSpBb–*Sau3AI* with HMSpAa). A first round of PCR was carried out with splinkerette primer HMSp1 and PB transposon primers PB5′-1 or PB3′-1. Then 1% of the PCR product was directly used for a second-round nested PCR, which was carried out with splinkerette primer HMSp2 and the PB transposon primers PB5′-2 or PB3′-2. Primers for splinkerette-PCR and sequencing are shown in Supplementary table S1. The nested PCR products were purified by High Pure 96 UF cleanup plate (Roche) and were used for sequencing with primer PB5′-seq or PB3′-seq separately.

### Southern blotting and hybridization

Total genomic DNA was restricted; size fractionated on agarose gels, blotted, and hybridized using standard procedures. The following probes were used: *LacZ* probe, a 800 base pair *Cla*I digested fragment from pSAgeo [14], which is a plasmid containing the *SA*-β-*geo* cassette in pBlueScript.

### Genomic long range and triple primer PCR

Genomic long range PCR (LR-PCR) was performed with primer pairs representing the genomic sequences flanking the PB gene-trap insertion (supplementary table S1). Using the recommended PCR conditions (Thermo), the PCR product of homozygous ES cell clones incorporates the 6.6 kb PB gene-trap cassette whilst heterozygous clones only have a small 428 bp genomic region.

Triple primer PCR was performed with primer pairs representing the genomic sequences flanking the PB gene trap insertion and one PB end primer (Supplementary table S1). The PCR reaction was competitive between the amplifications from the genomic sequences and the PB primer and genomic primer. The wild type, heterozygous or homozygous ES cell clone have different but comparable size PCR products.

### PBase reversal

Five million cells were electroporated with 20 µg PiggyBac transposase (PBase) expression plasmid, plated on 90 mm feeder plates, and 2 days later the cells were re-plated at low density (1,000 cells/10 cm plate) to allow the growth of individual ES cell colonies. Clones were picked into 96-well plates and the deletion of the PB transposon was confirmed by PCR. Ricin resistance was assessed by plating10^5^ cells/well in 24-well plates, exposing to ricin for 3 days and 6 days further culture with normal medium. Cells were visualized by staining with 2% methylene blue as described previously.

### Lectin screen

Kits of fluorescein isothiocyanate (FITC) conjugated lectins, encompassing several sugar specificities, were purchased from Vector Laboratories. Cells were harvested by trypsinisation and re-suspended at 1×10^6^/ml in staining buffer (1× Dulbecco's (D)-PBS containing 5% foetal calf serum and 0.01% sodium azide). Aliquots of 100 µl were incubated with 2 µl (1–2 mg/ml) lectin for 20 m at 4°C prior to extensive washing with D-PBS. After fixation in 100 µl 1% paraformaldehyde in D-PBS, stained cells were analysed by flow cytometry on the FL-1 green channel of a Becton Dickinson FACsAria.

### Glycosylation inhibitors

The inhibitors deoxynojirimycin, deoxymannojirimycin and swainsonine (VWR) were diluted to 100 mM in sterile D-PBS as stock solutions. Further dilutions of 1, 10 and 100 µM [15] were prepared in either normal media, for pre-treatment, or in medium containing 10 pM ricin. AB2.2 or NN5 cells, plated at 0.5×10^5^/well in 24 well plates one day earlier, were pre-treated, in duplicates, with inhibitor for initially 4 h at 37°C prior to ricin exposure. The inhibitor was then removed and replaced with fresh medium containing either inhibitor and/or 10 pM ricin or ricin alone. The cells were exposed for 3 days before neutral red staining. Due to the nature of ES cell growth in that domed colonies form and not monolayers, pre-treatment was extended to 24 hours in subsequent experiments to ensure full exposure to the inhibitors.

### Glycomics analysis

Pellets of 2×10^6^ cells were washed once in PBS prior to freezing at −80°C. Corresponding aliquots of the relevant mediums were also frozen at the same time. Glycoproteins and glycosphigolipids (GSLs) were extracted and separated by homogenisation and solvent partition ([29]. Cells were homogenised in 4 volumes of ice-cold water, the total aqueous volume was determined and glycolipids were extracted from the homogenate, by addition of 2.67 volumes of methanol and 1.33 volumes of chloroform, and vortexed thoroughly before centrifugation at 3000 rpm for 10 min. The glycoproteins were pelleted, reduced and carboxymethylated. After dialysis and lyophilisation, samples were digested with trypsin (Bovine TPCK treated T-1246, Sigma, Poole, UK) as previously described [30] and glycopeptides/peptides were purified on SepPak cartridge C18 (Waters Corp, Elstree, UK) [30]. The glycopeptides/peptides were dissolved in 200 µl of Ambic buffer (50 mM, pH 8.4). 3 U of PNGase F (1365177, Roche Applied Science, Burgess Hill, UK) was added and the reaction was incubated at 37°C for 24 hr, adding a fresh 3 U aliquot of enzyme after 12 hr. The digested samples were lyophilised and redissolved in 200 µl of 5% (v/v) acetic acid before being loaded onto a preconditioned Classic C18 Sep-Pak® and eluted with 5 ml of 5% (v/v) acetic acid (the N-glycan fraction) followed by elution with 4 ml of 20% and 40% (v/v) propan-1-ol in 5% (v/v) acetic acid (peptide/O-linked glycopeptide fractions). The 5% (v/v) acetic acid and combined 20% and 40% (v/v) propanol fractions were lyophilised. Permethylation and sample clean-up were performed using the sodium hydroxide protocol, as described previously [30]. Combined 20% and 40% (v/v) propanol fractions (peptide/O-linked glycopeptide fractions) resulting from the post-PNGase F digest were dissolved in 800 µl of 1 M potassium borohydride (KBH4) in a 0.1 M solution of potassium hydroxide (KOH) and incubated at 45°C for 20–22 hr. The reaction was terminated by drop wise addition of glacial acetic acid (5 drops). Cationic salts, amino acids and peptides were removed from the sample using a Dowex (50W-X8 (H+) 50–100 mesh) column. The O-glycans fractions obtained were then permethylated using the same method as the one used for the N-glycans.

After extraction of the pellet of glycoproteins, the supernatant containing the GSLs was transferred to a fresh tube and water (0.173 volumes of supernatant) added. After mixing and centrifugation (1550× g, 10 min), two phases were obtained. The lower phase was extracted twice with water and dried under nitrogen. The upper phase was cleaned-up on a C18 Plus Sep-Pak cartridge (Waters, Elstree, UK) as described earlier [29]. GSLs were incubated with ceramide glycanase (rEGCase II, 4460, Takara, Shiga, Japan). The reaction mixture was extracted with 1-butanol and the aqueous phase cleaned-up on a C18 Sep-Pak column. The glycans in the unbound fraction were purified on Hypercarb Cartridges (Waters, Elstree, UK) and freeze-dried. Glycans were deuteroreduced by 10 mg/mL sodium borodeuteride in 2 M ammonia solution (2 h, 25°C). After neutralization with acetic acid, the borates were removed by co-evaporation with 10% (v/v) acetic acid in methanol. Permethylation of glycans was performed using the sodium hydroxide procedure [31].

MALDI-TOF MS and MALDI-TOF/TOF MS/MS data were acquired using a 4800 MALDI TOF/TOF (Applied Biosystems) mass spectrometer. The MS spectra were obtained using the reflector positive mode with delayed extraction. MS/MS data were acquired using collision energy of 1 kV, and argon was used as collision gas. Samples were dissolved in 10 ml of methanol and mixed at a 1∶1 ratio (v/v) with 2,5-dihydrobenzoic acid as matrix.

### Immunogold staining of ricin and transmission electron microscopy

Cells incubated with 20 pM ricin for 1 h were fixed with 2% paraformaldehyde/0.2% glutaraldehyde in PBS, at 37°C for 10 minutes and then transferred to ice. The cells were rinsed three times with PBS and briefly quenched with 0.1% glycine in PBS at 37°C, covered with 1 ml of 1% gelatin, scraped with a freshly cut Teflon strip into a 1.5 ml Eppendorf and centrifuged at 3,000 rpm. The resulting pellet was gently re-suspended in 10% gelatin and centrifuged again before chilling at 4°C. Small chopped pieces were infiltrated with 2.3 M sucrose on a roller at 4°C overnight and then mounted on aluminium stubs and plunge frozen in liquid nitrogen. 60 nm ultrathin cryosections were cut on a Leica EM FC6 ultra microtome and labelled with rabbit anti-ricin toxin A chain (Abcam) followed by 10 nm protein A gold (University of Utrecht) following the protocol of Tokayasu [32], [33]. Sections were imaged on an FEI 120 kV Spirit Biotwin equipped with a F415 CCD camera.

## Supporting Information

Figure S1 **MALDI-TOF MS profiling of AB2.2, NN5 and F10 N-glycans.** N-glycans were permethylated prior being subjected to MS analysis and m/z values correspond to [M+Na]^+^ ions. For clarity reason, not all the peaks are labelled on these spectra but the m/z value and the annotations of all detected peaks are shown in Supplementary Figure 2. Grey boxes highlight the two regions detailed in Figures 7 and 8.
(TIF)

Figure S2 **Assignments of the molecular ions observed in the N-glycan profiles of AB2.2, NN5 and F10 cells.** Scheme assignments are based on the precise fit between composition calculations and the m/z (z = 1) ratio of the molecular ions detected. Ions with m/z in bold red are specific to F10 cells only. Schemes represent the most likely structures taking into account the biosynthetic pathways and the enzyme repertoire of murine cells and selected MS/MS analyses.
(TIF)

Table S1 **Splinkerette, sequencing and PCR primers.** Details of all splinkerette, sequencing and PCR primers utilised to generate vectors and confirm gene trap mutations.
(DOC)

Table S2 **Assignments of molecular ions [M+Na]^+^ observed in the MALDI-TOF MS spectra of reduced and permethylated O-glycan derived from AB2-2, NN5 and F10 cells. NeuAc: N-Acetylneuraminic acid, Hex: Hexose, HexNAc: N-Acetylhexosamine.**
(DOC)

Table S3 **Assignments of molecular ions [M+Na]^+^ observed in the MALDI-TOF MS spectra of deuteroreduced and permethylated glycosphingolipids derived from AB2-2, NN5 and F10 cells.**
(DOC)
