# Supplementary figures and images for: Mannosidase 2, alpha 1 Deficiency Is Associated with Ricin Resistance in Embryonic Stem (ES) Cells
Source: PLoS One. 2011 Aug 23;6(8):e22993. doi: 10.1371/journal.pone.0022993 (PMC3160287; doi:10.1371/journal.pone.0022993)

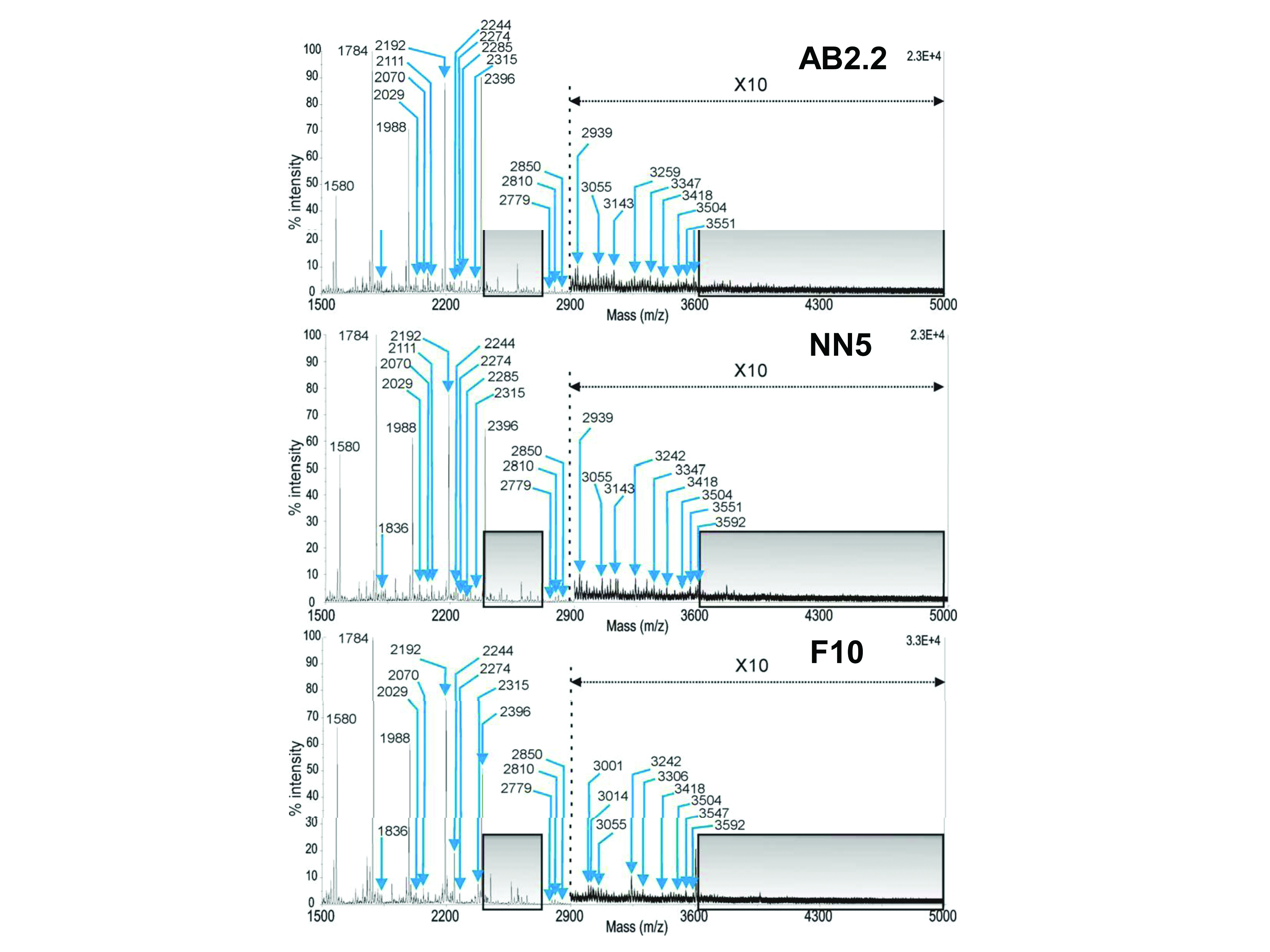

Supplement: Figure S1 — MALDI-TOF MS profiling of AB2.2, NN5 and F10 N-glycans. N-glycans were permethylated prior being subjected to MS analysis and m/z values correspond to [M+Na]+ ions. For clarity reason, not all the peaks are labelled on these spectra but the m/z value and the annotations of all detected peaks are shown in Supplementary Figure 2. Grey boxes highlight the two regions detailed in Figures 7 and 8. (TIF) [file pone.0022993.s001.tif]

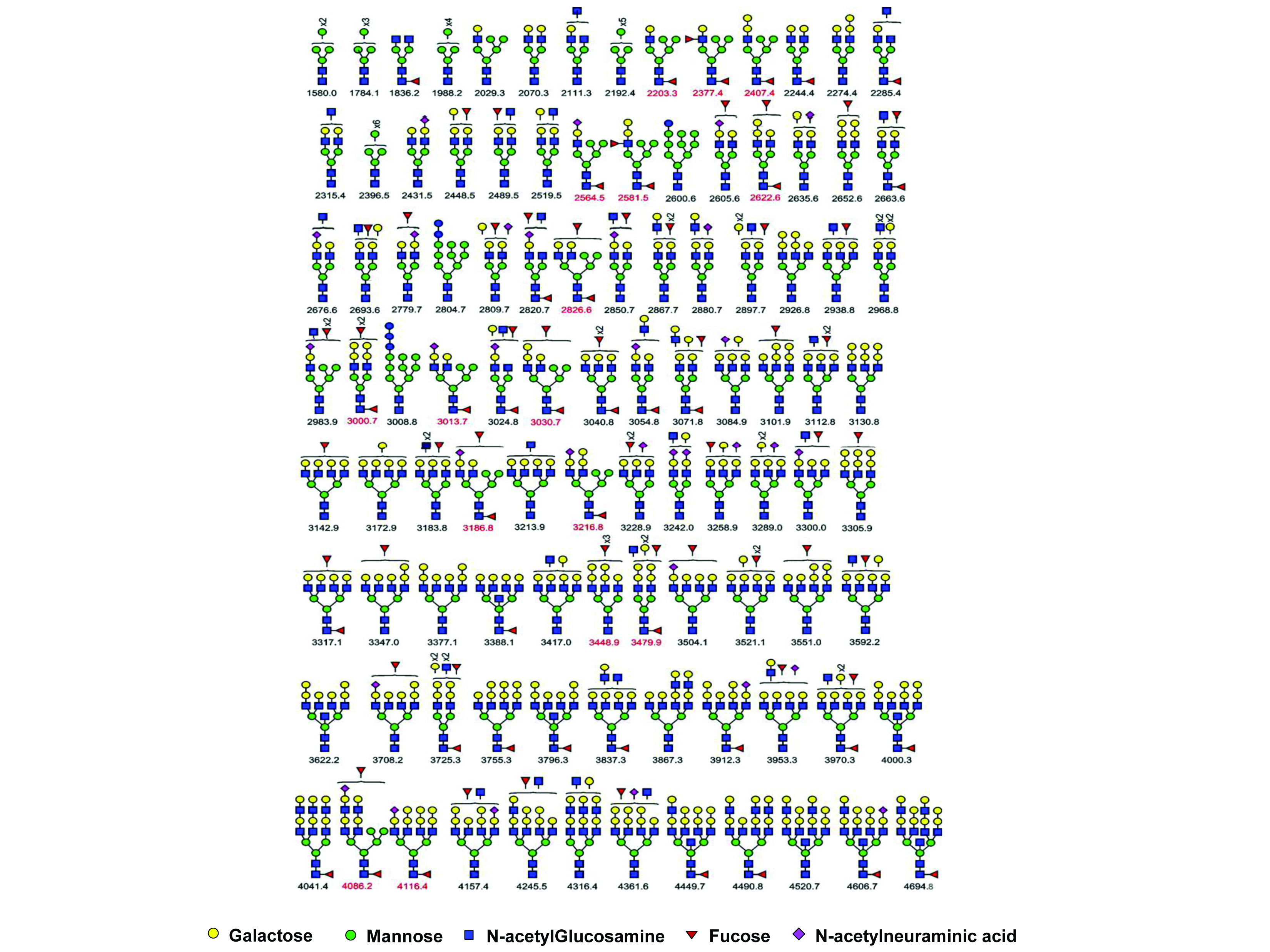

Supplement: Figure S2 — Assignments of the molecular ions observed in the N-glycan profiles of AB2.2, NN5 and F10 cells. Scheme assignments are based on the precise fit between composition calculations and the m/z (z = 1) ratio of the molecular ions detected. Ions with m/z in bold red are specific to F10 cells only. Schemes represent the most likely structures taking into account the biosynthetic pathways and the enzyme repertoire of murine cells and selected MS/MS analyses. (TIF) [file pone.0022993.s002.tif]
