# Supplementary material for: Mannosidase 2, alpha 1 Deficiency Is Associated with Ricin Resistance in Embryonic Stem (ES) Cells
Source: PLoS One. 2011 Aug 23;6(8):e22993. doi: 10.1371/journal.pone.0022993 (PMC3160287; doi:10.1371/journal.pone.0022993)
Supplement: Table S1 — Splinkerette, sequencing and PCR primers. Details of all splinkerette, sequencing and PCR primers utilised to generate vectors and confirm gene trap mutations. (DOC) [file pone.0022993.s003.doc]

**Supplementary Table 1**

**Splinkerette, sequencing and PCR primers.**

Details of all splinkerette, sequencing and PCR primers utilised to generate vectors and confirm gene trap mutations.

| **Splinkerette-PCR** |  |
| --- | --- |
| HMSpAa | 5’--CGAAGAGTAACCGTTGCTAGGAGAGACCGTGGCTGAAT  GAGACTGGT GTCGACACTAGTGG--3’ |
| HMSpBb-Sau3A1 | 5’--GATCCCACTAGTGTCGACACCAGTCTCTAATTTTTTTTTT  CAAAAAA--3’ |
| HMSp1 | 5’--CGAAGAGTAACCGTTGCTAGGAGAGACC--3’ |
| HMSp2 | 5’--GTGGCTGAATGAGACTGGTGTCGA--3’ |
| PB5’-1 | 5’--TAAATAAACCTCGATATACAGACCGATAAA--3’ |
| PB5’-2 | 5’--ATATACAGACCGATAAAACACATGCGTCAA--3’ |
| PB5’-seq | 5’--TTTTACGCATGATTATCTTTAACGTACGTC--3’ |
| PB3’-1 | 5’--CAAAATCAGTGACACTTACCGCATTGACAA--3’ |
| PB3’-2 | 5’--CTTACCGCATTGACAAGCACGCCTCACGGG--3’ |
| PB3’-seq | 5’--TTAGAAAGAGAGAGCAATATTTCAAGAATG--3’ |
| **Genomic Primers** |  |
| Man2a1-exon21-up | 5’--ATGGGCAAAGGCTATTCGGATGAGGCAGCC--3’ |
| Man2a1-exon22-down | 5’--GAAGCTAAAGTAATGTGCTTAGTTATTGCA--3’ |
