# Supplementary material for: Mannosidase 2, alpha 1 Deficiency Is Associated with Ricin Resistance in Embryonic Stem (ES) Cells
Source: PLoS One. 2011 Aug 23;6(8):e22993. doi: 10.1371/journal.pone.0022993 (PMC3160287; doi:10.1371/journal.pone.0022993)
Supplement: Table S2 — Assignments of molecular ions [M+Na]+ observed in the MALDI-TOF MS spectra of reduced and permethylated O-glycan derived from AB2-2, NN5 and F10 cells. NeuAc: N-Acetylneuraminic acid, Hex: Hexose, HexNAc: N-Acetylhexosamine. (DOC) [file pone.0022993.s004.doc]

**Supplementary Table 2**

**Assignments of molecular ions [M+Na]+ observed in the MALDI-TOF MS spectra of reduced and permethylated O-glycan derived from AB2-2, NN5 and F10 cells. NeuAc: N-Acetylneuraminic acid, Hex: Hexose, HexNAc: N-Acetylhexosamine**

| **m/z of [M+Na]+ ion** | **Composition** |
| --- | --- |
| 895.5 | NeuAc1Hex1HexNAc1 |
| 983.6 | Hex2HexNAc2 |
| 1187.7 | Hex3HexNAc2 |
| 1256.7 | NeuAc2Hex1HexNAc1 |
| 1344.8 | NeuAc1Hex2HexNAc2 |
