# Supplementary material for: Mannosidase 2, alpha 1 Deficiency Is Associated with Ricin Resistance in Embryonic Stem (ES) Cells
Source: PLoS One. 2011 Aug 23;6(8):e22993. doi: 10.1371/journal.pone.0022993 (PMC3160287; doi:10.1371/journal.pone.0022993)
Supplement: Table S3 — Assignments of molecular ions [M+Na]+ observed in the MALDI-TOF MS spectra of deuteroreduced and permethylated glycosphingolipids derived from AB2-2, NN5 and F10 cells. (DOC) [file pone.0022993.s005.doc]

**Supplementary Table 3**

**Assignments of molecular ions [M+Na]+ observed in the MALDI-TOF MS spectra of deuteroreduced and permethylated glycosphingolipids derived from AB2-2, NN5 and F10 cells.**

| **m/z of [M+Na]+ ion** | **Composition** |
| --- | --- |
| 855.5 | NeuAc1Hex2 |
| 943.5 | Hex3HexNAc1 |
| 1100.6 | NeuAc1Hex2HexNAc1 |
| 1147.6 | Hex4HexNAc1 |
| 1216.1 | NeuAc2Hex2 |
| 1304.7 | NeuAc1Hex3HexNAc1 |
| 1665.8 | NeuAc2Hex3HexNAc1 |
| 2027.1 | NeuAc2Hex3HexNAc1 |
